# Bridging the Gap between Glycosylation and Vesicle Traffic

**DOI:** 10.3389/fcell.2016.00015

**Published:** 2016-03-08

**Authors:** Peter Fisher, Daniel Ungar

**Affiliations:** Department of Biology, University of YorkYork, UK

**Keywords:** Golgi apparatus, glycan processing, COG complex, congenital disorders of glycosylation, vesicle tethering

## Abstract

Glycosylation is recognized as a vitally important posttranslational modification. The structure of glycans that decorate proteins and lipids is largely dictated by biosynthetic reactions occurring in the Golgi apparatus. This biosynthesis relies on the relative distribution of glycosyltransferases and glycosidases, which is maintained by retrograde vesicle traffic between Golgi cisternae. Tethering of vesicles at the Golgi apparatus prior to fusion is regulated by Rab GTPases, coiled-coil tethers termed golgins and the multisubunit tethering complex known as the conserved oligomeric Golgi (COG) complex. In this review we discuss the mechanisms involved in vesicle tethering at the Golgi apparatus and highlight the importance of tethering in the context of glycan biosynthesis and a set of diseases known as congenital disorders of glycosylation.

## Introduction to glycosylation

Glycans are a universal feature in cell biology, the process of glycosylation covers proteins and lipids with often elaborate carbohydrate chains. Mammalian cells contain approximately 200 glycan processing enzymes capable of modifying carbohydrate chains. The processing of such chains is essential for many different developmental and cellular processes, for example early mammalian development (Ioffe and Stanley, 1994; Shi et al., 2004; Ye and Marth, 2004; Grasa et al., 2012). Protein glycosylation is categorized as *N*-linked if the glycan is attached to the amide nitrogen of an asparagine residue, or O-linked if the bond is between the glycan and the oxygen of a serine or threonine sidechain. This review will focus on the relationship between *N*-glycan biosynthesis/function and the process of vesicle tethering at the Golgi apparatus.

**Figure 1:**
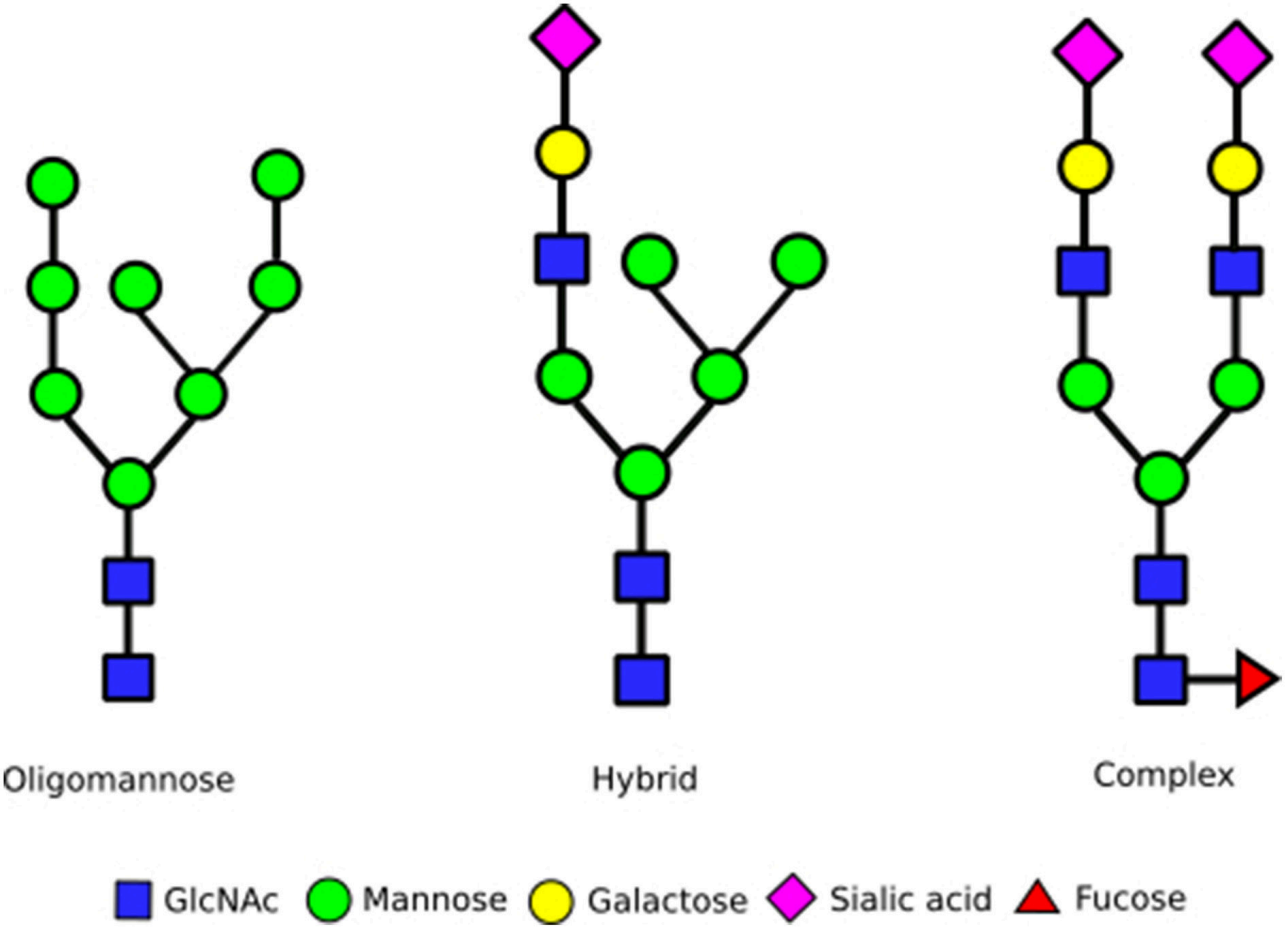
**Different stages of ***N***-glycan complexity**. *N*-linked glycans are classified into oligomannose, hybrid and complex type glycans based on their structures. Hybrid and in particular complex *N*-glycans can contain more branches than shown by the addition of extra GlcNAc residues onto the mannoses already functionalized this way. In addition, a GlcNAc can also be added to the mannose linked to the chitobiose core. This can result in upto five branches on complex glycans. Every *N-*glycan consists of a core built up of a chitobiose core [two *N-*acetyl glucosamines (GlcNAc)] that links to an asparagine sidechain in an Asn-X-Ser/Thr sequence, followed by three mannoses that initiate two separate branches. The antennae of *N*-glycans then consist of either all mannose residues (oligomannose glycans, left structure), a combination of GlcNAc, galactose, sialic acid and fucose residues (complex glycans, right structure) or a mixture of these with one branch being complex, the other oligomannose (middle structure).

*N*-glycan biosynthesis begins in the ER with the *en bloc* transfer of a 14 monosaccharide carbohydrate chain from dolichol to the nascent protein. Subsequent trimming of glucoses aids quality control during protein folding prior to ER exit. The resulting oligomannose glycan, containing eight or nine mannoses attached to a chitobiose core, undergoes processing into complex or hybrid forms at the Golgi apparatus (Figure 1). Complex glycan chains are composed of several *N-*acetylglucosamine (GlcNAc) seeded branches extended with the addition of galactose and sialic acid residues. The addition of GlcNAc residues can give rise to bi-, tri-, tetra- and penta-antennary complex glycan structures. In contrast, hybrid structures contain one or more complex branches alongside ain oligomannose branch. Glycosylation is inherently heterogeneous due to the competition of the various enzymes during glycan processing. Yet the exact distribution of *N*-glycan structures—referred to as the glycan profile—can have significant effects on cellular processes. For example, an impaired supply of CMP-sialic acid and GDP-fucose, which altered glycan processing in the Golgi, resulted in reduced protein secretion and increased ER stress in HeLa cells (Xu et al., 2010). So how do cells control the range and variety of glycan structures synthesized?

**Figure 2:**
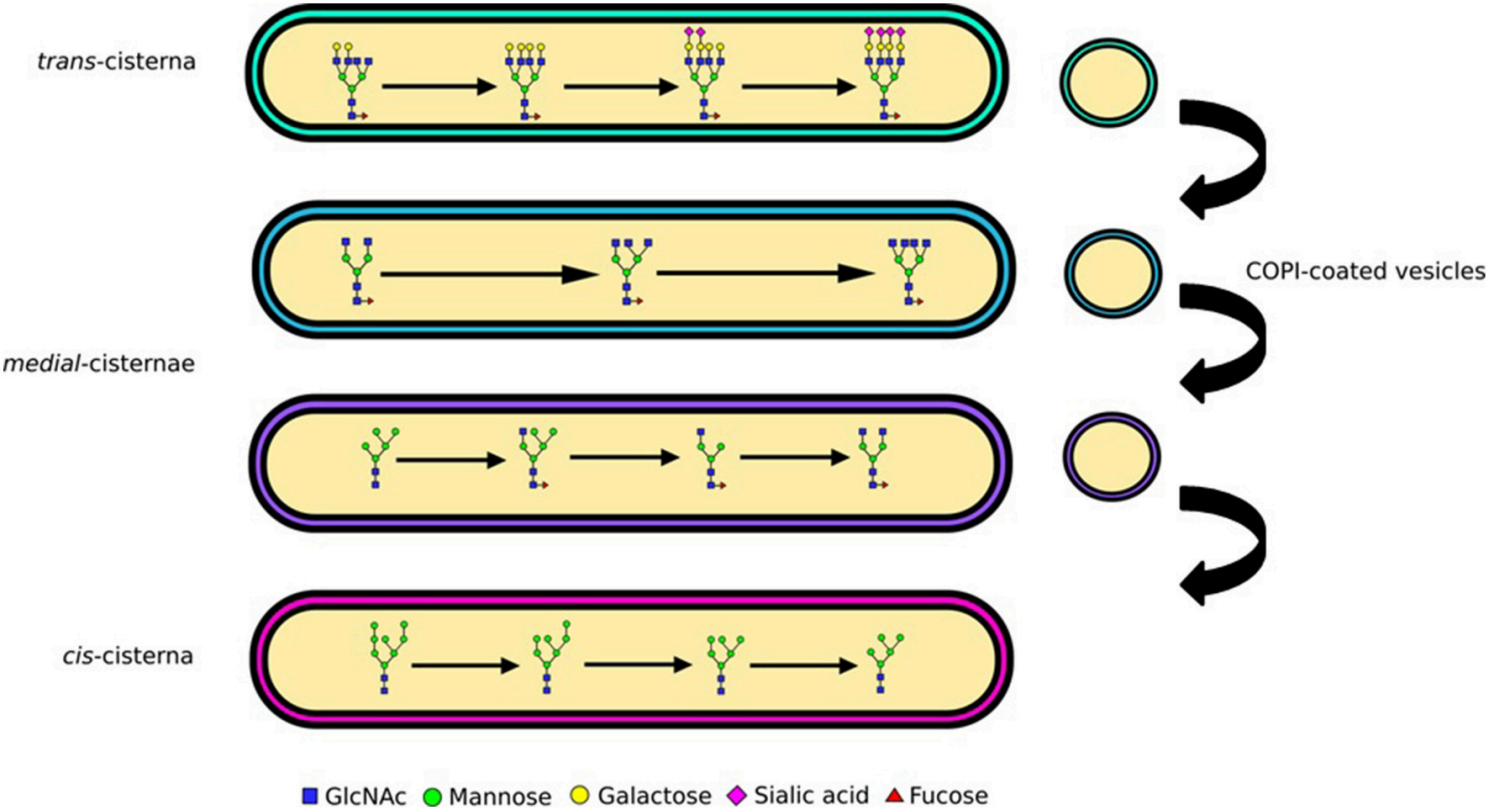
**Glycan processing and complexity**. *N*-linked glycans are processed from oligomannose to complex as they traverse the Golgi apparatus. The enzymatic reactions needed for processing of glycans from oligomannose to hybrid and complex are compartmentalized into Golgi cisternae. Mannose trimming enzymes are in the *cis* and *medial* Golgi, GlcNAc addition and associated branching in the *medial*, while capping with galactose and sialic acid in the *trans*. The differential distribution of these enzymes is maintained through vesicular sorting, with COPI-coated vesicles moving them in the retrograde direction.

The Golgi is sub-compartmentalized into several cisternae (Figure 2), each containing a different subset of glycosylation enzymes (Rabouille et al., 1995; Dejgaard et al., 2007). As secretory proteins traverse the Golgi from the *cis* to the *trans* side they are sequentially subjected to these enzyme subsets (Kornfeld and Kornfeld, 1985; Stanley, 2011). Mannose trimming occurs in the *cis* cisternae followed by the addition of GlcNAc residues and further mannose trimming in the *medial* cisternae (Figure 2). The antennae of *N*-glycans are finally extended and capped with the addition of galactoses and then sialic acids (Figure 2). Yet, the biosynthesis pathway gains additional complexity through the competition of various enzymes, which also provides unanticipated pathways to the particular *N*-glycan profiles. For example, although glycan branching is initiated by GlcNAc-transferases (GlcNAcTs) prior to galactose addition, the activity of galactiosyltransferase-4 (iGalT-4) was found to be a major regulator for the production of *N*-glycans with multiple antennae (McDonald, 2014). The diversity of a cell's glycan profile is dictated by two factors: how much of each enzyme is expressed in the cell, and where these enzymes are located within the Golgi. The fact that these two factors are complementary for determining glycan diversity was nicely illustrated in a study that compared glycan profiles and glycosylation enzyme transcriptome data in stem cells and embryoid bodies derived from them. Only a third of the changes in the transcriptome and glycome correlated during the cellular differentiation process studied, the rest did not (Nairn, 2012). This shows that enzyme expression is only one parameter encoding the glycan profile, and implying key importance for enzyme localization.

To preserve the functionality of glycosylation the precise distribution of enzymes involved in glycan biosynthesis must be maintained as secretory cargo passes through the Golgi apparatus. Both the retention of enzymes amidst secretory protein flow and their sorting into distinct cisternae can be explained by the cisternal maturation model of Golgi transport (Papanikou and Glick, 2014). This model postulates that glycosylation enzymes travel in retrograde vesicles that target to specific cisternae (Figure 2). Targeting specificity is likely maintained by distinct but overlapping protein machinery that ensures tethering and subsequent fusion of the vesicles with the cisternal membranes (Cottam and Ungar, 2012).

## Enzyme sorting at the golgi apparatus

Disruption to the retrograde sorting of enzymes, will affect the enzymatic makeup of Golgi cisternae, and thereby profoundly alter cellular glycan profiles. How this sorting and spatial partitioning of enzymes into cisternae occurs has been subject to much debate. It is known that certain physiological conditions, such as the slightly acidic pH of the Golgi, are required for normal glycosylation. The existence of a pH gradient through the Golgi stack invites the notion that each cisterna is an optimized reactor for a subset of enzymes (Gawlitzek et al., 2000). However, pH changes between adjacent cisternae are unlikely to be this drastic. Alternatively, an altered pH could cause incorrect localization of glycosyltransferases, leading to a loss of ability to execute reactions in the correct order (Rivinoja et al., 2009; Maeda and Kinoshita, 2010). In support of a riole of pH in enzyme sorting, mutations to a subunit of the vesicular H^+^-ATPase, which is involved in acidification of the Golgi, have been found to impair glycosylation. This results from slowed retrograde trafficking, as evidenced by a delayed transport of Golgi residents to the ER, and causes glycosylation disorders in patients (Kornak, 2008). Mislocalization of glycosyltransferases upon Golgi pH neutralization with a weak base or an ATPase proton pump inhibitor has also been observed (Axelsson et al., 2001). A possible pH dependent mechanism to regulate trafficking is through the formation of enzyme oligomers, which depends on cisternal pH (Hassinen and Kellokumpu, 2014). These enzyme oligomers are also important for precise and efficient glycosylation.

A further contributing factor to enzyme sorting is the membrane composition and thickness of each cisterna (Patterson et al., 2008). Indeed, the localization of several Golgi resident proteins to individual cisternae was altered when the level of sphingomyelin, which influences membrane thickness, was changed (van Galen et al., 2014). A comprehensive analysis of transmembrane domains (TMDs) in the secretory pathway has uncovered a strong correlation between TMD lengths and the *cis* to *trans* distribution of resident Golgi proteins (Sharpe et al., 2010). The pH-dependent oligomerization and TMD length of enzymes are two important determinants of Golgi localization. Additional factors, which are not related to enzyme sorting, such as cellular nucleotide sugar concentrations, and steric accessibility of the glycosylation site and the glycan chain itself will also undoubtedly play a role in determining *N*-glycan structure but will not be discussed further. Next we will consider the mechanisms contributing to the trafficking of enzymes, which ultimately provides the machinery for sorting.

Coatomer (COPI) coated carriers at the Golgi have long been implicated in the retrograde transport and sorting of Golgi-enzymes (Figure 2). A recent study has, however, highlighted the role of the GTPase Cdc42 in regulating COPI mediated transport directionality (Park et al., 2015), providing further evidence that under appropriate circumstances COPI carriers can traffic in anterograde as well as in the retrograde direction. This may resolve some of the previous debates on the content of COPI carriers containing predominantly anterograde or retrograde cargo (Orci et al., 2000; Gilchrist et al., 2006). The mechanism of enzyme packaging into intra-Golgi COPI carriers, in particular motif driven sorting of Golgi-residents, remains elusive. Some information came from the observation that numerous Golgi resident proteins where mislocalized in yeast upon deletion of Vps74p (Schmitz et al., 2008), which was therefore suggested to act as a sorting receptor. Although there is little sequence conservation between glycosyltransferases, short motifs containing basic residues in the cytoplasmic tails of enzymes where indeed shown to interact with Vps74p and its mammalian homolog GOLPH3 (Tu et al., 2008; Banfield, 2011; Eckert et al., 2014). Other sequences containing basic residues have been found in mammalian enzymes and are required for Golgi retention, although the interactions of these with the GOLPH3 and COPI machineries have not been tested (Uemura et al., 2015). Another recently identified sorting factor for enzymes is keratin-1, which was shown to be essential for the Golgi localization of the C2GnT-M enzyme (Petrosyan et al., 2015). Although the mechanism by which it acts is not known, it could be involved in enzyme scaffolding, sorting of proteins into COPI carriers, or the targeting of the carriers themselves, all novel functions for intermediate filament proteins. But what are the known molecular players involved in targeting COPI carriers and their cargo to the correct cisternae?

## Molecular players in golgi vesicle tethering

Vesicle targeting uses the factors responsible for tethering and subsequent membrane fusion. The initial contact between the destination membrane and vesicle, known as tethering, is where the decisions about targeting specificity are most likely made. However, proteins of the fusion machinery, in particular SNAREs, are also likely involved in the targeting process. Therefore, an important contributor to the blueprint for cellular glycan profiles is encoded within the protein interactions of the vesicle tethering and fusion machineries. These are the interactions, which will ultimately deliver Golgi enzymes to their respective cisternae.

### SNAREs and SM proteins

Soluble NSF attachment protein receptors (SNAREs) provide the driving force necessary to fuse two membranes together, but the assembly of a fusogenic SNARE complex also provides some of the targeting specificity (McNew et al., 2000). Fourteen SNAREs are localized to the Golgi apparatus, but only binding between cognate sets of SNAREs is capable of promoting efficient fusion. Other combinations of SNAREs have been shown to form complexes but were unable to promote membrane fusion (McNew et al., 2000). Fusogenic SNARE complexes may act as the final error check in vesicle targeting. Alternatively, protein interactions of individual SNAREs with tethering factors may recruit and facilitate the formation of SNARE complexes, providing a targeting system for COPI vesicles, as has been shown in the COPII trafficking pathway (Bentley et al., 2006). In line with this latter idea, several of the Golgi SNAREs interact with the conserved oligomeric Golgi (COG) tethering complex as detailed below.

The role of the Sec1/Munc18 (SM) family of proteins in SNARE mediated fusion has been long recognized. SM proteins are known to interact predominantly with the syntaxin (Stx) family of SNAREs (Misura et al., 2000), but SM protein binding to the whole SNARE complex has also been observed (Carr et al., 1999; Togneri et al., 2006; Lobingier and Merz, 2012). For the Golgi localized SM protein, Sly1, the main type of reported binding was to the N-terminus of Stx5 (Yamaguchi et al., 2002), a binding mode that is consistent with a role in SNARE complex formation (Kosodo et al., 2002; Peng and Gallwitz, 2002). This role for Sly1 has now been confirmed in a reconstituted *in vitro* system (Demircioglu et al., 2014). Most recently, a possible mechanism of SM protein mediated SNARE complex formation was revealed by a detailed structural characterization of SNARE-SM protein complexes. The yeast SM protein Vps33 was shown to bind simultaneously to two SNARE proteins forming a half-zippered SNARE complex, indicating templated folding of the four helical bundle on the SM protein (Baker et al., 2015).

### Rab GTPases and golgins

Small Rab GTPases play an active role in vesicle trafficking. Exchange of GDP for GTP initiates a conformational change allowing Rabs to recruit specific effectors for participation in vesicle trafficking. Subsequently, GTPase activating proteins promote conversion to the inactive GDP-bound Rab, which is released into the cytosol, relinquishing the attached effectors (Hutagalung and Novick, 2011). A handful of Rabs coordinate trafficking at the Golgi apparatus. One possible function at the Golgi would be in specifying cisternal identity, although more evidence will be needed to very this role (Pfeffer, 2013). For example, bidirectional transport of cargo at the Golgi apparatus requires both Rab6 and myosin II, with myosin II acting as a Rab6 effector (Miserey-Lenkei et al., 2010). Rab30 is found throughout the Golgi stack (Kelly et al., 2012) and has been shown to interact with the *Drosophila* orthologs of GM130 and GMAP-210 (Sinka et al., 2008; Gillingham et al., 2014). Further interactions of mammalian Rab30 with Cog4 have also been demonstrated (Fukuda et al., 2008; Miller et al., 2013). Another Golgi localized Rab, Rab33b has been shown to regulate retrograde *trans*- to *cis*-Golgi traffic of Shiga toxin B through recruitment of Rab6 suggesting a possible cascade-like mechanism of traffic regulation (Starr et al., 2010).

**Figure 3:**
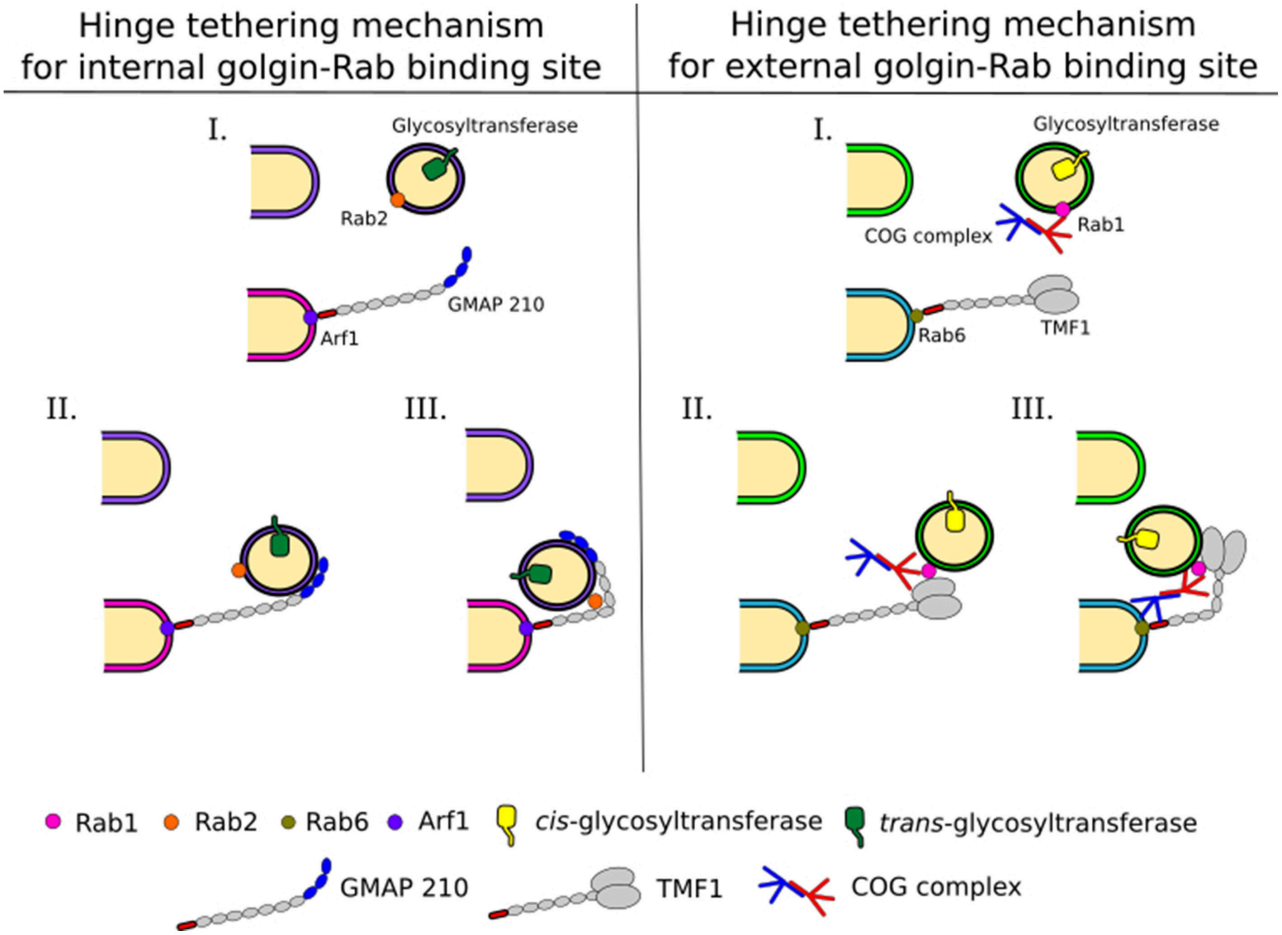
**Intra-Golgi retrograde vesicle tethering and targeting**. Vesicles carrying glycosylation enzymes are targeted to the correct cisternae by different combinations of interactions between trafficking proteins, including Rabs, golgins, and the COG complex. This allows for a complex targeting system leading to the maintenance of enzyme localization within the Golgi, which is pertinent to glycan processing. Golgins grab vesicles at a long distance from the cisternal membranes. This tethering and the movement of the vesicle to the cisternal membrane at the base of the golgin are assisted by Rabs and COG. Possible mechanisms for these are depicted and explained in the text.

Coiled-coil tethers that localize to the Golgi are referred to as golgins and may extend several hundred nm in length. Golgins have distinct localizations in the Golgi stack, for example, some contain GRIP domains and associate with Arl GTPases at the *trans* Golgi (Setty et al., 2003). The related GRAB domain of GMAP-210 binds to Arf1 at the *cis-*cisterna (Drin et al., 2008). Alternatively, the golgin TMF is recruited to the Golgi through its interaction with Rab6 (Fridmann-Sirkis et al., 2004). Due to their extended lengths golgins are ideal to tether vesicles far from their targets (Figure 3). Combined with a selective recognition system for vesicles of distinct cargo content golgins could provide a reliable targeting system used for maintaining glycosylation homeostasis in the Golgi (Wong and Munro, 2014).

Interactions of golgins with other trafficking factors, such as the COG complex, as discussed below, will be needed to bring the vesicle into close proximity of the target (Figure 3). For this to occur, breaks within the coiled-coil regions of golgins, which allow conformational changes to bring transport vesicles within close range of target membranes could be important (Cheung and Pfeffer, 2015). In case of GMAP-210 the presence of a Rab2 binding site within the coiled-coil region is postulated to hinge the golgin to facilitate vesicle docking (Sato et al., 2015, Figure 3). A hinging mechanism for TMF involving the COG complex was also proposed (Miller et al., 2013). For more details on golgin function the reader is referred to another review in this topical series (Witkos and Lowe, 2016).

### Conserved oligomeric golgi complex

**Figure 4:**
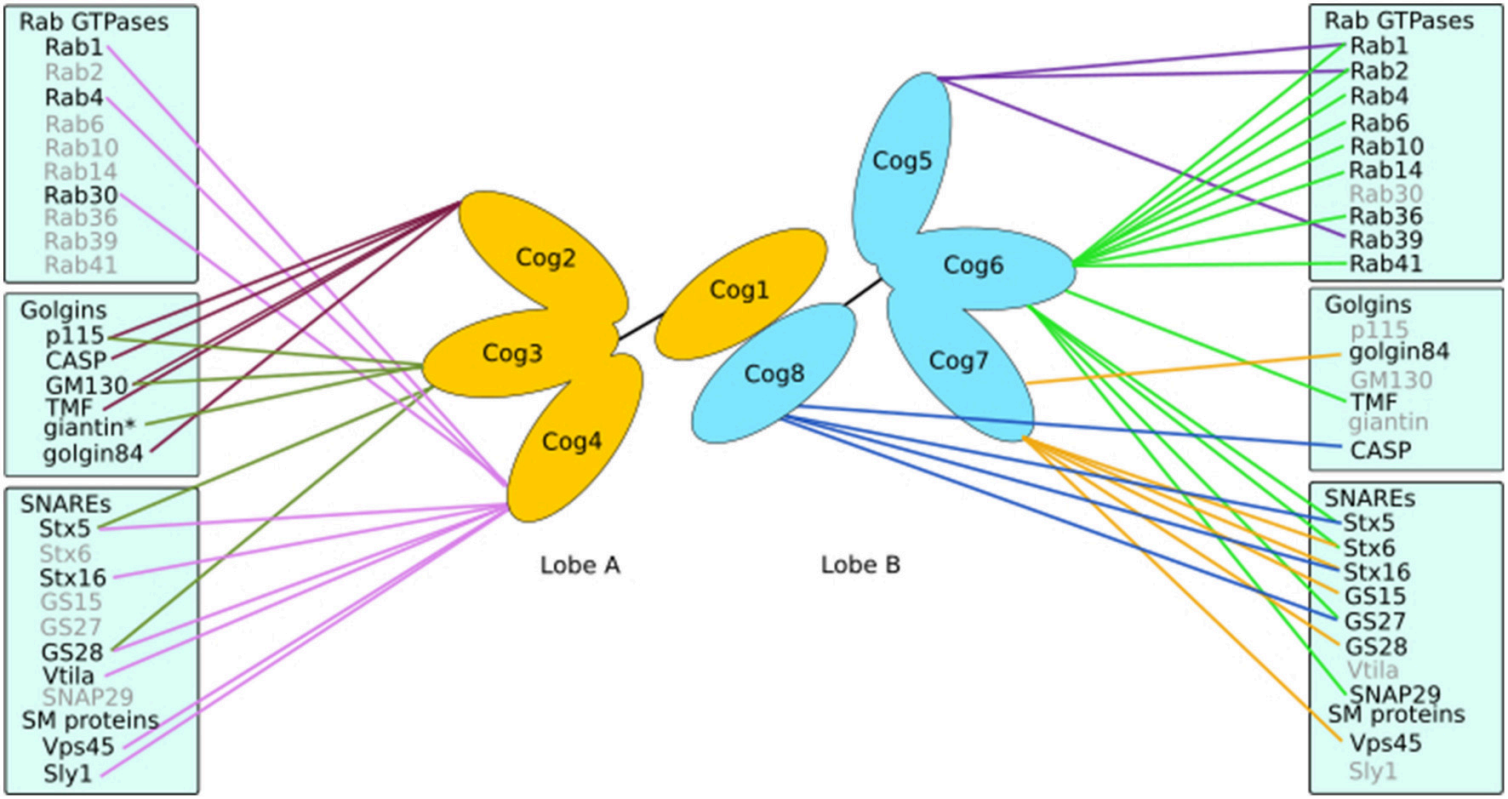
**Interactions of the COG complex**. The COG complex forms a bi-lobed structure with subunits Cog1-4 making up lobe A and lobe B consisting of Cog5-8. Each subunit (except Cog1) has been demonstrated to interact with numerous proteins involved in Golgi trafficking and tethering such as golgins, SNAREs and Rabs. ^*^Indicates an interaction with the COG complex as a whole.

The COG complex is a multi-subunit tethering complex and a member of the CATCHR (complexes associated with tethering containing helical rods) family of proteins (Yu and Hughson, 2010). It is a hetero-octamer with a tentacular layout of subunits (Ungar et al., 2005; Lees et al., 2010), which appears well suited to a role in vesicle tethering (Miller and Ungar, 2012). The eight subunits of the COG complex may be separated into two lobes (Figure 4), lobe A (containing Cog1-4) and lobe B (containing Cog5-8). A number of interactors of the COG subunits have been identified that are involved in Golgi trafficking, including SNAREs, Rabs, golgins, and vesicle coat proteins (Willett et al., 2013b; Figure 4). Importantly, defects in COG have been associated with glycosylation abnormalities in all studied model organisms as well as humans (Kingsley et al., 1986; Suvorova et al., 2002; Struwe and Reinhold, 2012). Moreover, the integral membrane protein TMEM115, which is enriched in the Golgi, interacts with the COG complex, while its knockdown has adverse effects on glycosylation (Ong et al., 2014).

There is evidence that COG subunits may regulate membrane fusion by promoting SNARE complex formation and discouraging the formation of non-fusogenic SNARE complexes *in vivo* (Hong and Lev, 2014). However, if this reflects a property of COG to regulate SNARE trafficking, or to directly influence SNARE complex formation will need further investigation. It is clear though that trafficking selectivity is an important role for COG, as Cog4 was capable of recruiting Stx5 associated vesicles *in vivo*, in contrast to Cog8, which attracted Stx16 carriers. This demonstrated the ability of the COG complex to differentiate between various intra-Golgi vesicles (Willett et al., 2013a). Indeed, several COG subunits, including Cog4, Cog6, and Cog8 have been found to directly interact with a number of SNAREs, for example Stx5, Stx6, Stx16, GS27 and SNAP29 (Kudlyk et al., 2013; Willett et al., 2013a; Figure 4). The fact that Cog4 was able to pull down Stx16 and components of both Stx5 and Stx16 containing SNARE complexes (Laufman et al., 2013b) could well be due to the efficient capture of the whole complex in both reactions. The COG complex could also help coordinate SM proteins and SNAREs. This is supported by the interaction between Cog4 and Sly1 adjacent to Cog4's Stx5 binding site (Laufman et al., 2009; Willett et al., 2013a). Such interactions suggest a functional role of the COG complex in linking tethering and fusion events through assisting SNARE complex assembly and/or correctly localizing cognate SNAREs and SM proteins.

Interactions between mammalian COG subunits and GTP-bound Rabs have been demonstrated for Rab1, Rab2, Rab4, Rab6, Rab10, Rab14, Rab30, Rab36, Rab39, and Rab41 (Fukuda et al., 2008; Miller et al., 2013; Figure 4), implicating the COG complex as a *bona fide* Rab effector. The functional role of COG-Rab interactions remains speculative. Possibilities are that the complex is recruited to domains or vesicles defined by particular Rabs, which results in targeted tethering and ensuing enzyme sorting. Alternatively, or in addition, the COG-Rab interactions could play a mechanistic role in vesicle docking as well (Miller et al., 2013; Figure 3).

Finally, the COG complex also interacts with golgins (Figure 4). Every golgin so far investigated has been found to interact with the Cog2 subunit (Sohda et al., 2007, 2010; Miller et al., 2013). Yet, most golgins also interact with other COG subunits in a combinatorial pattern. For example, golgin84 interacts with Cog7 (Sohda et al., 2010), GM130 with Cog3 and Cog5, TMF with Cog6 and CASP with Cog8 (Miller et al., 2013). Interestingly, the ability of the COG complex to interact with both ends of the golgin TMF could enable that the complex in conjunction with Rab1 and Rab6 promotes tighter docking of a tethered vesicle (Miller et al., 2013, Figure 3).

## Targeted tethering for correct glycosylation

From the above discussions it should not come as a surprise that intra-Golgi vesicle tethering has a strong influence on the fidelity of glycosylation. The following section will highlight specific examples of this. A nice relationship between Golgi tethering and glycosylation is provided by the *Drosophila melanogaster* sugar-free–frosting (sff) gene. This is a Golgi localized homolog of the SAD-1 kinase, which itself promotes vesicle tethering at *C. elegans* synapses. Sff mutations caused a defect in the expression of neural specific *N*-linked glycans, concomitant with a defect in vesicle tethering at the Golgi (Baas et al., 2011). Another connection is phosphorylation of the Golgi reassembly stacking protein GRASP65, which has been linked to Golgi fragmentation, and accompanying glycosylation abnormalities in Alzheimers disease (Joshi et al., 2014). Together with GRASP55, GRASP65 has also been shown to regulate protein trafficking and the expression of complex *N*-glycans under physiological conditions (Xiang et al., 2013). An important function of GRASP proteins is the lateral fusion of cisternae from adjacent Golgi stacks, a process that is needed to maintain homogeneous enzyme distributions in cisternae upon ribbon formation (Puthenveedu et al., 2006; Jarvela and Linstedt, 2014). It is therefore not unexpected that GRASPs are essential for glycan homeostasis.

**Figure 5:**
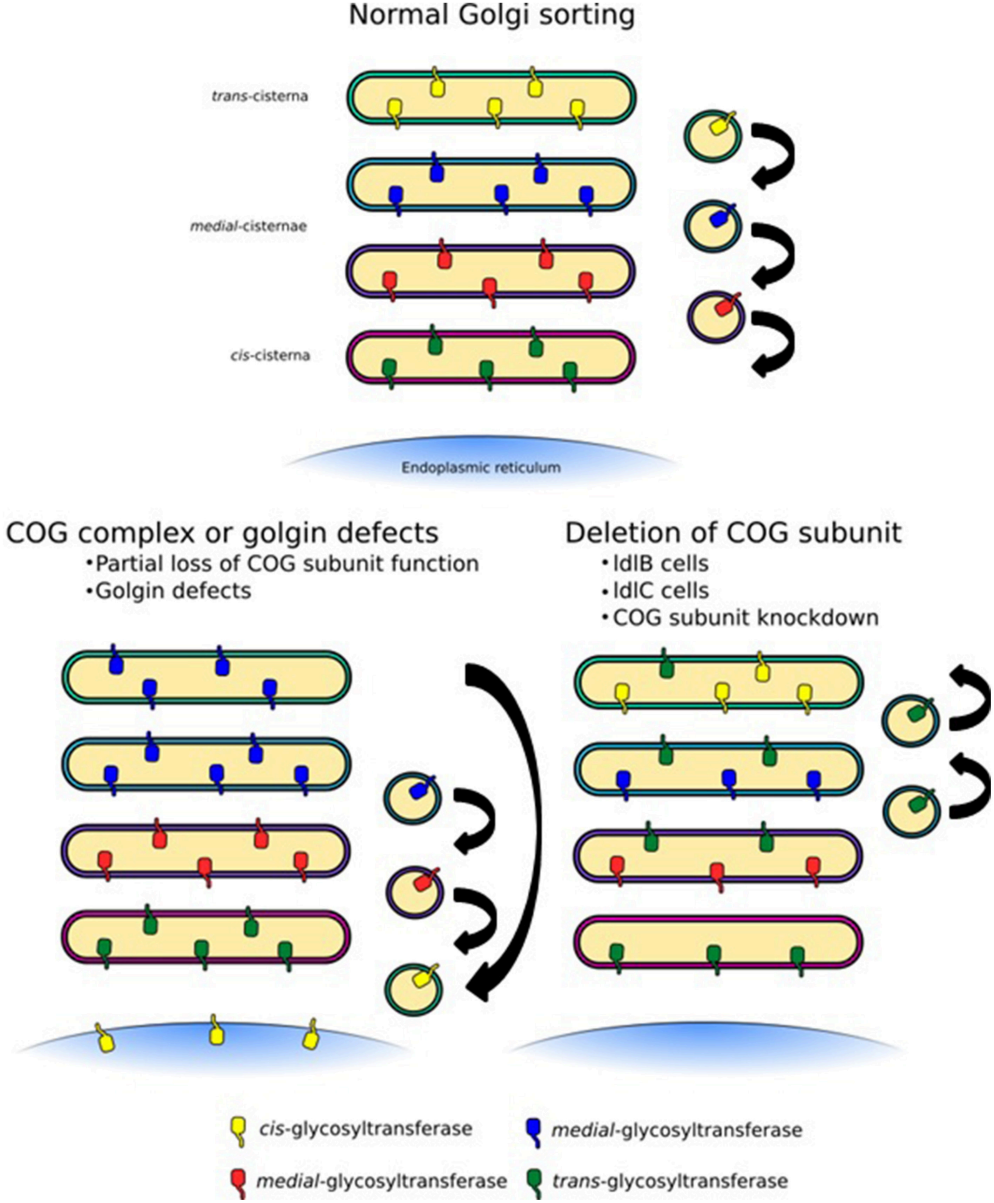
**Trafficking defects at the Golgi apparatus**. Normal Golgi trafficking, as predicted by the cisternal maturation model, in which COPI-vesicles transport enzymes in the retrograde direction to sort them to their respective cisternal destinations. Different forms of trafficking defects can manifest themselves in a number of ways. COG-CDGs and golgin mutations are likely to lead to the mislocalization of a specific subset of cargo containing vesicles depending on the COG subunit or golgin that is affected (left side). In this case some enzymes, for example sialyltransferases, could be lost from cisternae. More global glycosylation defects, which would likely be embryonically lethal but can be observed in tissue culture, may be the result of an unstable COG complex as a whole, for example due to deletion of a full subunit (right side). This would lead to the loss of several enzymes, but could also broaden the distribution of some enzymes, such as mannosidase I.

Golgins have been shown to tether specific vesicles (Malsam et al., 2005), suggesting they play a role in a sophisticated targeting operation (Figures 3, 5). For example, giantin knockdown mislocalized the glycosyltransferase C2GnT-M, whereas GM130 knockdown prevented Golgi targeting of C1GalT1 (Petrosyan et al., 2012), and TMF knockdown displaced GalNAc-T2 from its correct Golgi location (Yamane et al., 2007). A recent study has shown that vesicle targeting is, at least in part, encoded in the golgin proteins. Ectopic expression of TMF was capable of relocating GalNAcT-2 carriers to mitochondria, whereas golgin84 and GMAP-210 efficiently recruited the *cis*-Golgi protein ZFPL1 to mitochondria (Wong and Munro, 2014). While golgins are clearly important for glycosylation enzyme sorting, the aberrant glycosylation observed in golgin84 knockdown cells may well be mediated by the COG complex. The COG-golgin84 interaction was shown to be necessary for SNARE complex formation, which may be the direct cause of the observed glycosylation abnormalities (Sohda et al., 2010). Alternatively, the mislocalization of untethered vesicles as a result of golgin84 depletion may also explain the glycosylation defects (Figure 5). The ability of golgins to maintain Golgi morphology can also be linked to glycosylation homeostasis. Depletion of giantin caused a decrease in stack size and with that an alteration in glycosylation (Koreishi et al., 2013).

The COG complex is central to intra-Golgi retrograde trafficking due to its ability to coordinate Rab GTPases, golgins, coat proteins and SNAREs during vesicle tethering. Many groups have used chemical methods or siRNA techniques to knockdown or knockout individual subunits of the COG complex. Such methods often lead to an unstable complex and as a consequence depletion of other subunits has been observed. This global effect not only drastically reduces the number of binding interactions that the COG complex can participate in but tethering events as a whole will be a rarer occurrence at the Golgi. Furthermore, if proteins required for the budding of vesicles are not efficiently recycled, budding events may also be inhibited. The implication of a system with this impaired tethering could lead to a dilution of mannosidase I throughout the Golgi stack with an apparent increase in oligomannose processing, but an overall decrease in complex glycan formation associated with a higher proportion of the Man_5_GlcNAc_2_ species (Figure 5). This trend has been observed in glycan profiling experiments on Cog1 and Cog2 mutant ldlB and ldlC CHO cells (Abdul Rahman et al., 2014). This is a very likely scenario in many other cell culture based COG disruption experiments, as *N*-glycan processing is not required for cell growth and survival, thus cells may be viable despite quite drastic alterations to glycan processing. However, these defects would almost certainly be embryonic lethal in an organism, as shown for Cog3 depleted fruit flies (Schnorrer et al., 2010). Hence it is milder mutations that are often found in clinical settings.

The COG complex has been shown to be necessary for the correct localization of several glycosylation enzymes, including MGAT1, MGAT2, MAN2A1, GALT, and ST6GAL1 (Shestakova et al., 2006; Pokrovskaya et al., 2011). Glycan profiling has revealed decreased levels of sialylation in Cog3- and Cog4-knockdown HeLa cells, but slight increases in sialylation in Cog6- and Cog8- knockdown cells suggesting different roles for each lobe (Pokrovskaya et al., 2011). Indeed, lobe A of the COG complex is more essential for normal Golgi structure while lobe B, although more dispensable for this function, is required for maintaining the steady state levels of both GalT1-GFP and SiaT1-GFP (Peanne et al., 2010). Latter effect is possibly due to tethering defects of tr*ans-G*olgi vesicles, as the transport efficiency of GalT containing vesicles was adversely affected in a cell free tethering assay utilizing cytosol isolated from Cog6 or Cog7 deficient human fibroblasts (Cottam et al., 2014). As discussed above, in contrast to knockdown cells, the full absence of lobe A subunits in the ldlB and ldlC cells causes much more dramatic alterations (Abdul Rahman et al., 2014).

As mentioned, COG subunit mutations, knockdowns or deletions have knock-on effects on other COG subunits, their functional consequences are therefore often difficult to interpret. For example, the ldlC cells have an unstable COG complex with reductions in the levels of all COG subunits (Oka et al., 2005), which results in the inability to efficiently process glycans (Kingsley et al., 1986; Abdul Rahman et al., 2014). While the glycosylation phenotype of the Cog1 deficient ldlB cells is the same as that of ldlC cells (Abdul Rahman et al., 2014), it does not have altered levels of the other lobe A subunits (Oka et al., 2005). More precise incisions into the complex's architecture and protein interactions are needed to query its precise role in Golgi trafficking and ensuing glycan homeostasis. The disruption of the interaction between Cog5 and Cog7, for example, while it did not greatly perturb the stability of lobe B, had profound effects on Golgi trafficking and medial/late-Golgi glycan processing (Ha et al., 2014). Overall, the modification of COG function or a disorganized COG complex could result in either the complete abrogation of tethering in a particular Golgi region, or the introduction of a more subtle “fault” within the targeting system, which would lead to glycosyltransferase mislocalization (Figure 5). Given the overall developmental importance of glycans combined with the necessity for intra-Golgi trafficking for glycan homeostasis, it is evident that trafficking defects could well cause human glycosylation disorders.

## Trafficking and glycosylation disorders

The roles of glycan biosynthesis and function have been highlighted in a number of human diseases including cancer, inflammation and a set of genetic disorders known as congenital disorders of glycosylation (CDG). The mutations that cause CDG are generally hypomorphic and cause defects in glycan processing that lead to highly variable clinical manifestations (Hennet and Cabalzar, 2015). The molecular basis of *N*-glycan CDG ranges from defects in building and transferring the oligosaccharide precursor in the ER to defects that occur during glycan processing in the Golgi apparatus. The latter type of CDG may be due to faults in the glycosylation enzymes themselves. However, in line with the importance of glycosylation enzyme sorting, CDG can also be caused by defects in Golgi trafficking, which disrupts the delicate distribution pertinent to correct glycan processing (Wu et al., 2004).

Due to improvements in diagnostics and whole exome sequencing, the discovery rate of new CDG subtypes has been increasing over the past years. As part of this advance CDG causing mutations have been found in all but one COG subunit, Cog3 being the outlier (Miller and Ungar, 2012; Kodera et al., 2015). As the COG complex is implicated in the targeted tethering of Golgi glycosylation enzymes, COG-CDGs provide examples of how defects in intra-Golgi tethering can manifest themselves clinically through glycosylation defects. This is typically in a pleiotropic manner, impacting on multiple organs, as COG mutations cause alterations to *N*-, O- and lipid-linked glycosylation in all cell types. We will now discuss some examples of how tethering defects can cause human disease through the impairment of glycosylation. The most prominent features of all COG-CDG patients are incomplete sialylation and galactosylation. These can be seen with both lobe A and lobe B subunit mutations. However, while in lobe A these are without fail missense mutations or truncations (Foulquier et al., 2006; Reynders et al., 2009), in the case of lobe B subunits these can be full loss-of-function mutants (Wu et al., 2004; Paesold-Burda et al., 2009). This is in line with model organism studies, in which a full loss of a lobe A subunit is embryonic lethal (Schnorrer et al., 2010).

Missense mutations are found in CDG-Cog2 and -Cog4 patients. The mutation in the Cog2 subunit reduces the stability of other lobe A subunits resulting in an increase in monosialyated and agalacto transferrin species (Kodera et al., 2015). The integrity of all lobe A subunits and Cog5 in lobe B are all compromised as a result of a point mutation in the Cog4 gene (Reynders et al., 2009). One of the most common COG-CDGs is a truncation of the Cog1 subunit, which causes reduced levels of Golgi α-mannosidase II and β-1,4 GalT in the perinuclear regions (Foulquier et al., 2006). Analysis of the patient's serum demonstrated irregularities in sialylation and galactosylation of *N*-glycans, in addition to a decrease in sialic acid on mucin type O-glycans (Faid et al., 2007). A reason for the viability of such a rather severe mutation may be found from the analysis of ldlB cells, which lack Cog1 altogether but maintain normal levels of the remaining lobe A subunits (Oka et al., 2005). A similar molecular outcome results from a loss of Cog8 in a patient with two different mutations in this subunit. The two truncations lead to complete lack of Cog8 in patient fibroblasts, and a severe reduction in other lobe B subunit levels as well as Cog1. As for the Cog1 patient, the remaining lobe A subunits are sufficient to maintain early-Golgi glycan processing, with the main defect being in sialylation of both *N-* and O-glycans (Kranz et al., 2007). The impact of COG on coordinating SNAREs has been demonstrated with cells derived from this CDG-Cog8 patient showing the importance of COG as the main organizer of intra-Golgi retrograde vesicle tethering. The steady state levels of the Golgi associated SNAREs GS28 and GS15 were found to be reduced in both CDG patient derived cells and Cog8 depleted HeLa cells (Laufman et al., 2013a).

Lobe B subunits appear to tolerate increasingly severe mutations although missense mutations also occur. An exon skipping mutation in the Cog5 gene resulted only in a mild clinical phenotype despite sialylation defects in both *N*- and O-glycans (Paesold-Burda et al., 2009). Other Cog5-CDG patients presented with more serious symptoms, including severe mental retardation (Rymen et al., 2012). Some of these could well affect the Cog5-Cog7 interaction due to insertions or deletions, since disruption of this binding interface displayed aberrant glycosylation in HEK293 cells (Ha et al., 2014). While Cog5-CDGs can have strongly reduced Cog7 levels, a complete loss of Cog7 leads to even more serious symptoms, namely death in the first 3 months of life (Wu et al., 2004). Cellular analysis of the Cog7-CDGs showed disrupted recycling of a variety of Golgi resident proteins, such as giantin and the SNARE GS15 (Steet and Kornfeld, 2006). This again demonstrates the role of the COG complex in coordinating the molecular players involved in tethering and fusion not just enzymes.

A number of patients have been identified with mutations to Cog6 (Rymen et al., 2015), a subunit closely linked to the Cog5-Cog7 dimer within the complex (Ungar et al., 2005). Importantly, Cog6 has been shown to interact with several SNAREs, Rabs and the golgin TMF (Kudlyk et al., 2013; Miller et al., 2013). A missense mutation in a patient led to the loss of stability for other members of lobe B and a glycosylation deficiency that proved fatal shortly after birth (Lübbehusen et al., 2010). A second Cog6-CDG patient with the same G549V mutation of the Cog6 protein as the above case demonstrated hyposialylation of serum transferrin, but was not lethal (Huybrechts et al., 2012). This highlights the importance of modifier mutations in the rest of the patients' genomes for the ultimately observed phenotypes. Given the generally small number of patients for each mutation, cellular and/or animal models will be essential to tease out the molecular contributions of each mutation to the various glycosylation and organismal phenotypes.

Finally, COG mutations are not the only trafficking related defects causing CDG. Nonsense mutations that lead to the loss of GMAP-210 protein have recently been identified as the cause of skeletal dysplasia in mice and achondrogenesis type 1A in humans. Loss of the protein was shown to cause glycan processing defects in the Golgi of the affected mice (Smits et al., 2010). As already discussed, GMAP-210 is necessary for the selective tethering of vesicles at the Golgi as well as maintaining Golgi morphology, and the abnormal glycosylation observed in GMAP-210 deficient animals points toward a fundamental connection between targeted tethering, glycan processing and skeletal development.

## Bridging the Gap between tethering and glycosylation

This review has described numerous examples of how the tethering of Golgi derived vesicles dictates glycan structure. What is currently unknown and requires further investigation is which specific interactions are required for the delivery of a particular set of glycosyltransferases to their correct cisternae. This is not a trivial problem to address as in many clinical and laboratory cases a reduction in sialylation and galactosylation is observed upon disruption of the Golgi trafficking machinery, however this may not simply be the result of missorting of sialyltransferases or galactosyltransferases. In theory a reduction in galactosylation and sialylation could be the result of improper sorting of other upstream glycan processing enzymes. For example, any conversion of oligomannose to complex glycan necessitates the activities of mannosidase I, GlcNAcTI, and mannosidase II. The implication of the complexity of *N*-linked glycosylation, that a number of reaction pathways may lead to the same final glycan structure, is that although a general defect in glycosylation is often a result of perturbed trafficking, the enzymes that are missorted may differ from case to case.

Interestingly, a number of COG-CDGs are not the result of complete loss of function mutations but are missense mutations that change only one amino acid such as the G549V mutation in the Cog6-CDG patient (Lübbehusen et al., 2010). Single amino acid substitutions are more likely to have local effects through the alteration of a single interaction between a COG subunit and a specific binding partner either by weakening or strengthening the interaction. This may be as a result of an alteration to subunit secondary structure or through the replacement of a necessary interacting residue. It is therefore intriguing to speculate that the G549V mutation in Cog6 (a Rab and SNARE interaction hub) for example weakens or strengthens the interaction of Cog6 with a specific SNARE, such as Stx6 or a Rab GTPase (e.g., Rab6) but does not completely prevent tethering from occurring. Given that lobe B subunits provide a platform for the tethering of *trans*-Golgi vesicles (Willett et al., 2013a) it may not be unreasonable to surmise that vesicles containing sialyltransferases and galactosyltransferases are relocated in this case. In terms of glycosylation, the characteristic reduction in sialylation and galactosylation often seen in COG-CDG patients would emerge as a result while minimal change to the oligomannose processing would occur in contrast to the knockdown cell lines discussed above. A possible mechanism of vesicle mislocalization is thus through the defective formation of SNARE/golgin landmarks at a given Golgi cisterna (Willett et al., 2013a) leading to the mislocalization of glycosylation enzymes. Untethered COPI vesicles may relocate and fuse to the ER (Figure 5).

Defects to golgins also alter Golgi trafficking and glycan processing (Smits et al., 2010). Unlike the COG complex, which is involved in the majority of tethering events at the Golgi, GMAP-210 is likely to regulate the tethering of only one or maybe a few types of specific cargo containing vesicles. Hence the glycosylation phenotype in GMAP-210 depleted cells may resemble that of sorting defects observed in COG mutations rather than subunit deletions (Figure 5). As a mechanism for GMAP-210 regulated tethering has been proposed (Sato et al., 2015, Figure 3) one can speculate about the impact of individual mutations on vesicle sorting and glycosylation. For instance, mutations to the GRAB domain may result in the relocation of the golgin to a different cisterna. This ectopic GMAP-210 would still be capable of tethering vesicles through its ALPS motif and Rab2 binding site meaning mislocalization of the original GMAP-210 targeted vesicle cargo. In this case it is likely that *cis*-Golgi enzymes such as mannosidase I and/or GlcNAcTI will be found in later cisternae of the Golgi stack given the ability of GMAP-210 to recruit the *cis*-Golgi protein ZFPL1 to mitochondria (Wong and Munro, 2014). If this is the case an elevation in the proportion of oligomannose and hybrid *N*-glycans would occur. Mutations to the Rab2 binding site or the ALPS motif in contrast, are likely to alter the identity of the targeted vesicle thereby recruiting the incorrect glycosyltransferases, or merely mistargeting the cognate glycosyltransferases, resulting in a different type of aberrant glycosylation. To understand how sorting is regulated at the Golgi, individual interactions between the relevant trafficking players must be investigated in the context of enzyme localization in the future.

## Concluding remarks

Glycan processing at the Golgi apparatus is an essential requirement for numerous cellular functions. Perturbations to the targeted tethering of vesicles that contain glycan modifying enzymes can drastically alter the glycan profile of the cell. Defects in different players in Golgi tethering contribute differently to the glycan processing pathway. If these defects are not interfering with embryonic development they can lead to human disease cases classed as CDG. While most of the currently known trafficking related CDGs are due to mutations in the COG complex, mutations in other trafficking components will likely emerge as current exome sequencing efforts of patients are further pursued. A good example of this is the recently discovered connection between glycan processing and skeletogenesis in GMAP-210 mutant patients. The correct localization of glycosylation enzymes within the Golgi apparatus dictates glycan structure and therefore glycoprotein and glycolipid properties and function. A template for glycan structures, such as is found for DNA, RNA and proteins, does not exist, but detailed rules for glycan processing could well be encoded in a combination of enzyme expression and localization. A more detailed molecular understanding of the specificity of the vesicle tethering machinery that is capable of the targeted delivery of Golgi vesicles will therefore be critical for decoding glycan processing in the future. This could open up the investigation of more subtle functions of glycans whose synthesis could well be subject to spatial and temporal regulation within the Golgi during various developmental and disease states.

## Author contributions

DU and PF wrote the paper together.

### Conflict of interest statement

The authors declare that the research was conducted in the absence of any commercial or financial relationships that could be construed as a potential conflict of interest.

## Abbreviations

CDG: congenital disorders of glycosylation
CHO: Chinese hamster ovary
COG: conserved oligomeric Golgi
GalNAcT: *N*-acetylgalactosamine transferase
GalT: galactosyltransferase
GlcNAc: *N-*acetylglucosamine
GlcNAcT: *N-*acetylglucosamine transferase
SiaT: sialyltransferase
SM: Sec1/Munc18
SNARE: soluble N-ethylmaleiamide sensitive factor attachment protein receptor
TMD: transmembrane domain.
